# Transparent, self-cleaning, scratch resistance and environment friendly coatings for glass substrate and their potential applications in outdoor and automobile industry

**DOI:** 10.1038/s41598-021-00230-9

**Published:** 2021-10-20

**Authors:** Phool Shahzadi, Syeda Rubina Gilani, Bakht Bahadur Rana, Abdul Ghaffar, Akhtar Munir

**Affiliations:** 1 Glass and Ceramics Research Centre (GCRC), Pakistan Council of Scientific and Industrial Research (PCSIR), Labs. Complex, Lahore, Pakistan; 2 Department of Chemistry, University of Engineering and Technology, Lahore, Pakistan; 3 Department of Chemistry, SBA, School of Science and Engineering, Lahore University of Management Sciences, Lahore, Pakistan; 4 Department of Chemistry, University of Sialkot, Sialkot, Pakistan

**Keywords:** Chemistry, Nanoscience and technology

## Abstract

In this research work six novel combinations of Hydroxy Ethyl Meth Acrylate based copolymers have been synthesized and commercial titania, after activation was added by adopting simple strategy to manufacture super-hydrophobic, cost effective, transparent, antifogging, self-cleaning and antimicrobial coating on the glass sheet which will be helpful for outdoor and automobile windscreen. The super-hydrophobic covering was set up by dip covering procedure and coated specimen have been characterized for Wetting behaviour, transparency and SEM analysis. Likewise, the dependability of the coating was evaluated at conditions comparable strengthening at higher temperatures (4–400 °C), illumination by UV spectrum at basic and acidic limits, Results revealed that developed material has good adhesion with glass and shows transparency more than 97%, and water contact edge (CA) of 135 ± 2°. Furthermore, the covering displays astounding self-cleaning property. All the outcomes demonstrated that such kind of coatings could be used many modern level applications on automobile wind screen and glass-windows in building and other glasses where protection from UV radiation, anti-fogging and cleaning is required. Such type of coating material can also be used to preserve architectural work leather and other decoration and artwork. The graphical representation is given in Fig. 1.

## Introduction

**Figure 1:**
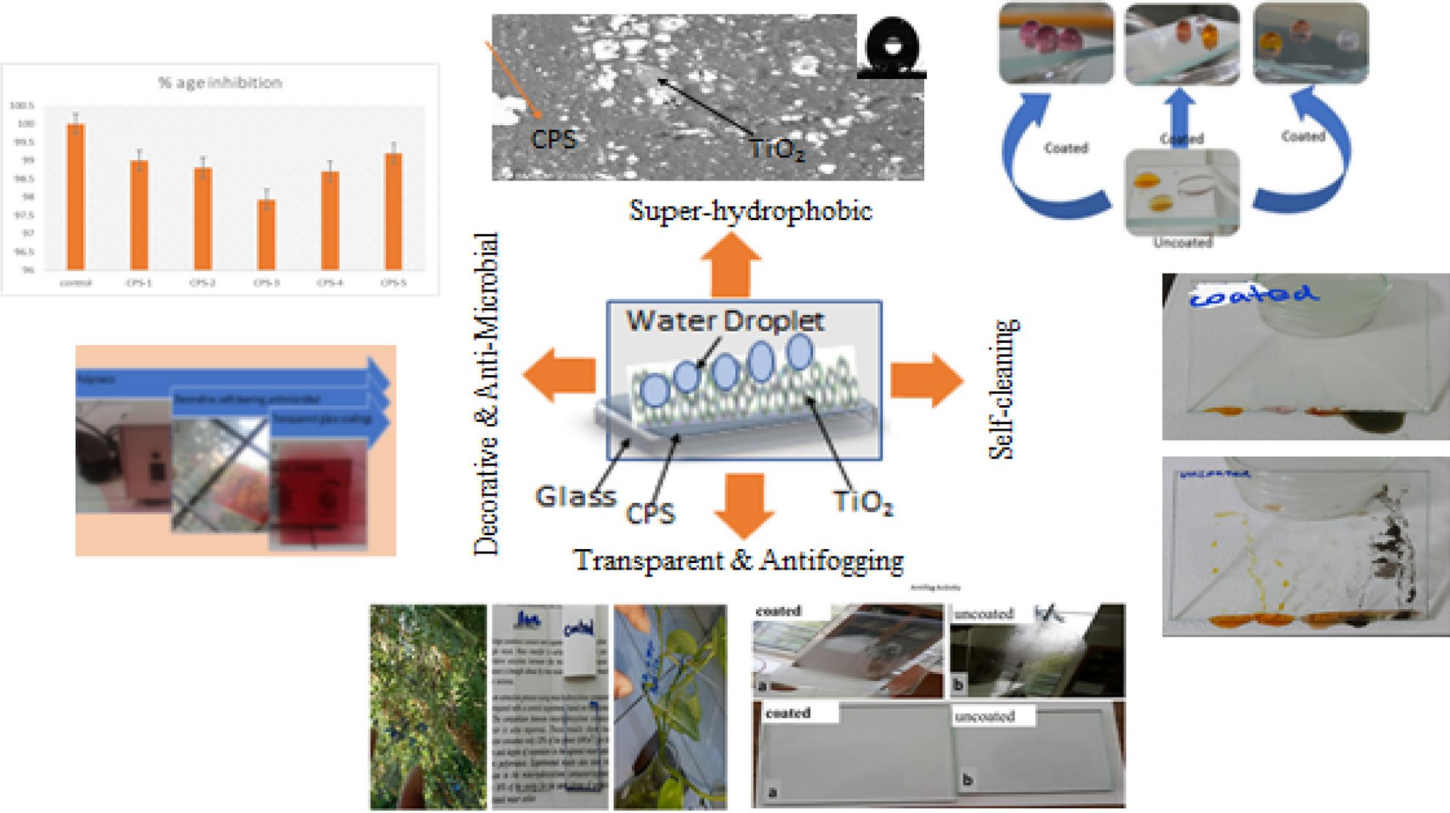
Graphical abstract.

Remembering the water emergencies in many nations and cleaning impact, it has gotten urgent to safe water for endurance of living creatures by utilizing less quantity of water for cleaning purposes. Widow cleaning with manual method is also very difficult for high buildings which look shabby at their exterior high in the sky and it includes human injury, wastage of time money and water. If we use self-cleaning coatings, it will be very helpful to save things from weathering and provide environment friendly atmospheric conditions. Likely uses of super hydrophobic surfaces for self-cleaning building outsides, window glasses, car windshields, and water-proof materials^1^. Super hydrophobic coatings have also received interest due to their anti-icing potential by reducing the time an impinging liquid droplet is in contact with the surface. Ice accumulation on airplanes is a significant hazard to human safety and the build-up of ice on car windshields is an annoyance in winter climates^2^. Super-hydrophobic surfaces observed widely on many plants leaves naturally^3,4^, insects epidermal layers and other extensions^5^. Such type of surface has ability of self-cleaning to remove contaminants on them by rolling off falling water drops on them. From an application view, super-hydrophobic surfaces will become particularly useful when several functions^6^ transparency, photocatalytic properties^7^ and self-healing after damage. Now a day, attention is diverted towards modified economic coatings for antimicrobial, photocatalytic and self-cleaning properties. Titanium dioxide (TiO_2_), for this purpose used in accordance with polymeric materials due to cost effective and its photocatalytic nature. Acrylic copolymers are used for coating purposes when titanium dioxide used along with acrylates it will provides better adhesion and good photocatalytic property^8,9^. So glass surface made activated by increasing roughness of surface and then by applied coated material by dip coating method. Smooth and activated surfaces experience different water contact angle due to change in surface energy^10^. The higher water contact angles is responsible for super-hydrophobic surfaces^11^. In geometry, angle below and above 90° referred to the hydrophilic and hydrophobic nature of exterior respectively^12^. And the limit of contact angle for hydrophilicity and hydrophobicity is ~ 65°. Henceforth, considering bond power, hydrophilic superficial suggests exteriors/surfaces having centres of the edge underneath 65°. whereas surfaces having angle more than 65° termed as hydrophobic exteriors^13^. Same is the case observed in Berg Limits based on the adhesion forces^14^. Super hydrophobic surfaces^15^, Lotus leaf like surfaces^16^ Superomniphobic surfaces provide effective chemical shielding^17^ and some other slippery and porous surfaces^18,19^. Polymeric coating material has excellent antimicrobial activity against *E Coli.*^20^. Fluorinated polymeric compounds also used for coating and coated surfaces has hydrophobic properties which increases its self-cleaning activity^21^. Porous silicon has been used to produce hydrophobic structures resulting in improvement of wettability^22^. Some coating materials has been developed with photocatalytic action on surfaces resulting in super-hydrophobicity^23^. Diatomaceous earth material used to fabricate coatings having super-hydrophobic action^24^. PDMS-Grafted-SiO_2_/TiO_2_ used to produce thin films that have multiple applications with photocatalitically stable super hydrophobicity^25^. The acrylate-based polymers were prepared by varying the nature and ratios of the monomers^26^ (Fig. 1).

## Experimental section

### Materials

**Figure 2:**
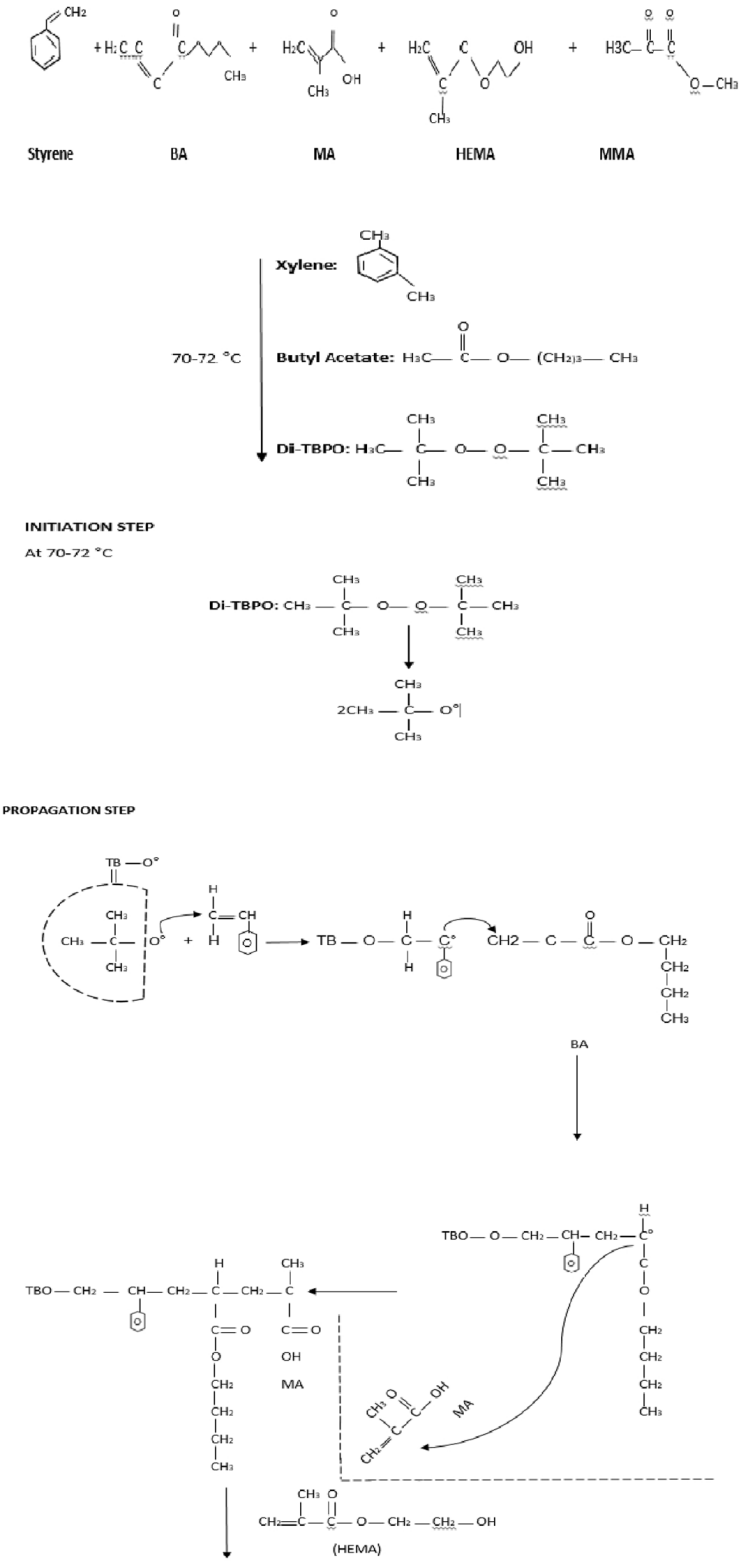

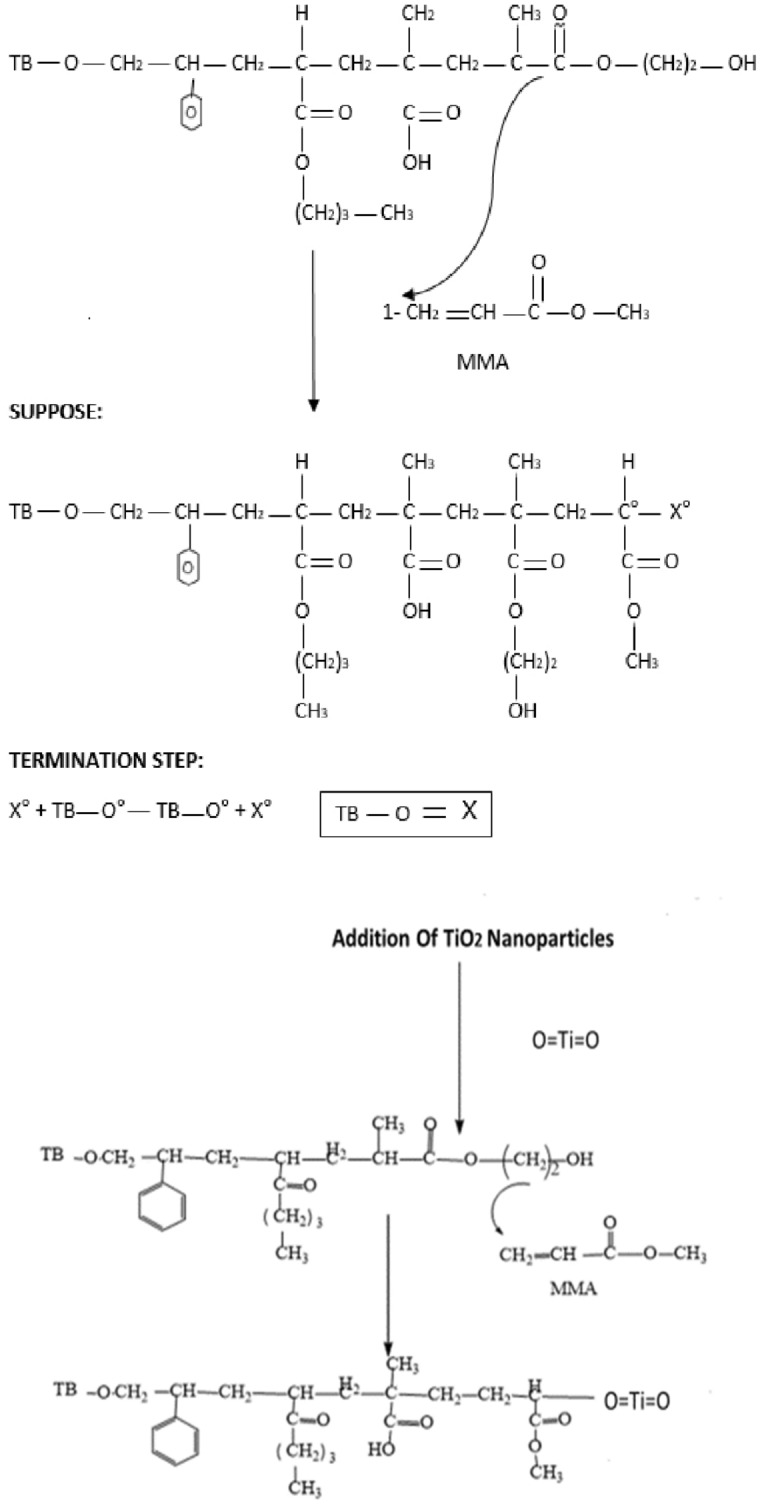
Reaction mechanism scheme.

The analytical grade chemicals including Xylene, Styrene, Methyl Meth Acrylate, Butyl Acrylate, Hydroxy ethyl Meth Acrylate (HEMA), Meth Acrylic Acid, Butyl Acetate and Di-Tertiary Butyl Per oxide) (used as catalyst) was purchased from Sigma Aldrich (Germany). The reaction mechanism is given in Fig. 2 and chemical composition in Table 1 given below;

**Table 1:** Composition of polymeric coatings.

Sr #	Materials	CPS-1 (wt%)	CPS-2 (wt%)	CPS-3 (wt%)	CPS-4 (wt%)	CPS-5 (wt%)	CPS-6 (wt%)
1	Xylene	33.9	34.3	33	33.55	31.9	30.9
2	Styrene	13.4	12.2	11	10.52	13.9	14.9
3	Methyl Meth Acrylate	26.4	27.4	25.8	25.8	27.3	26.3
4	Butyl Acrylate	13.4	13.3	16	15.99	13.2	14.2
5	Hydroxy ethyl Meth Acrylate (HEMA)	1.3	1.3	1.5	1.57	1.8	2.8
6	Meth Acrylic Acid	0.31	0.33	0.3	0.25	0.31	0.31
7	Butyl Acetate	11.5	11.3	12.4	12.28	11.7	10.7
	Total	100	100	100	100	100	100

Catalyst: Di.ter.butyl peroxide :(0.5%).

### Method

The chemical composition for CPS-1 to 6 is given in Table 1. The monomers added in reaction glass vessel fitted with 5 neck flange and a thermo-electric controlled unit to carryout polymerization, initiator Di-tertiary butyl peroxide was first dissolved in butyl acetate (Solvent) along with acrylate based monomers and polymerization was carried out at temperature of 80–90 °C followed by the addition of initiator by using dropping funnel at 5 equal intervals. After 3 h digestion was completed and a transparent copolymer product developed which is stored at room temperature for further dilution and addition of nano powder. Finally, developed product was preserved in steel and plastic jar and placed in refrigerator at 25–30 °C. Glass slides were cleaned with distilled water dried in an oven at 80–85 °C followed by isopropyl alcohol and with drier drying.

### Activation of titanium dioxide

Titanium dioxide nanoparticles activated by using stearic acid solution melted at 60 °C for two hours and then added into CPS copolymer solution and optimize conditions by adjusting temperature and concentration.

### Dilution of copolymer/activation of glass surface and coating on glass

25 wt/wt% dilution of developed copolymer was made by using solvent Xylene. Glass slides of 25.4 mm × 76.2 mm (1'' × 3'') was purchased from local market (made in china), cleaned and washed with distilled water and surface was activated by using iso-propanol. A 25% diluted copolymer solution in xylene was taken in a beaker and stirred for 10 min by using magnetic stirrer to get homogenous solution, then activated titanium dioxide has been added mentioned in Table 2 and stirred for 20 min. Finally, activated dried glass slide was dipped in this solution for 30–50 s, dried at room temperature for 24 h. the coated glass slide was tested for FTIR, contact angle, optical properties and SEM analysis and thermal property.

**Table 2:** Addition of TiO_2_ nanoparticles.

Sr #	Formulations	TiO_2_ Nanoparticles (%)		
1	CPS-1	0.5	0.1	0.2
2	CPS-2	0.5	0.1	0.2
3	CPS-3	0.5	0.1	0.2
4	CPS-4	0.5	0.1	0.2
5	CPS-5	0.5	0.1	0.2
6	CPS-6	0.5	0.1	0.2

## Results and discussions

### FTIR and UV absorbance spectra of modified TiO_2_ coatings

**Figure 3:**
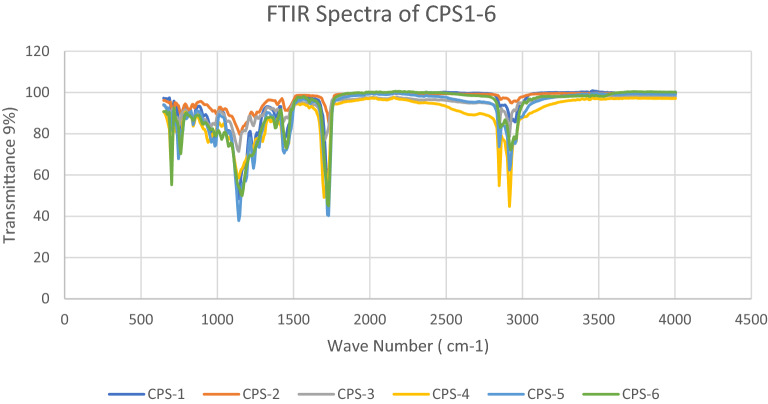
FTIR Spectra of CPS1-6.

**Figure 4:**
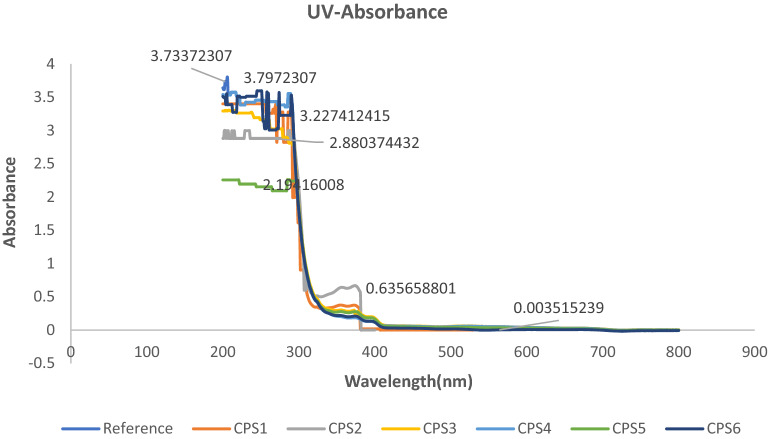
UV absorbance Spectra of CPS1-6.

FTIR analysis of modified acrylate copolymer showed the presence of TiO_2_. The Spectra shows little difference with presence of TiO_2_. The functionalization peaks seen in spectra due to absorption levels for Ti–O–C coordination. Following peaks have been shown 4000–3500 (not sharp peaks), OH stretching from HEMA, OH (Carboxylic Acids) 2952–3000 CH aliphatic and aromatic (peroxides in reaction mixture) and HEMA, 1750—1729 C=O from HEMA and Carboxylic derivative. 1470-1446 CH_3_,CH_2_ deformation,1237-1140 comes from C–O–C stretch from OH of HEMA and other polymer and peaks at 752-740 are due to Vibrational modes of TiO_2_, 780-400 are showing some crystalline titanium-dioxide as shown below in Fig. 3 and Table 3 given above. Bare glass and Coated material have been analysed for UV absorption and recorded absorbance in ultraviolet and visible region. Developed coating materials did not absorb UV radiation in visible region while show minimum absorbance in ultraviolet region which is clearly shown from graphs obtained from UV–Visible spectroscopy. Results revealed that there is only 3–5% absorbance which means that 97–95% light transmittance which is good sign for UV protection. The graphical data is given in Fig. 4.

**Table 3:** FTIR description of CPS1-6.

Sr. No	Wavenumber (cm^−1^)	FTIR Peaks
1	4000–3500	OH stretching from copolymer
2	3000–2952	CH aliphatic and aromatic (peroxides in reaction mixture) and HEMA
3	1750–1729	C=O From HEMA and Carboxylic derivative
4	1470–1446	CH_3_,CH_2_ deformation
5	1237–1140	C–O–C stretch from OH of HEMA and other polymer
6	984–879	C=CH_2_ from HEMA
7	752–740	Vibrational modes of TiO_2_ with copolymer
8	780–400	Crystalline TiO_2_

### Particle size analysis

**Figure 5:**
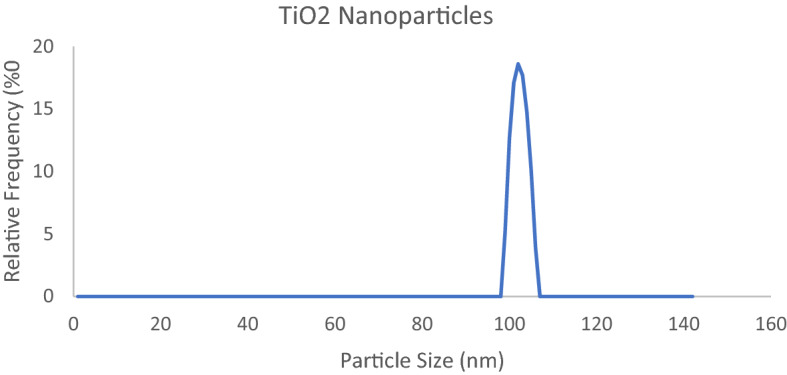
Particle size of TiO_2_ Nps.

The TiO_2_ nanoparticles used along with the synthesized polymeric mixture on glass slides were tested for size of particles, zeta potential determination and the results reported as follow Fig. 5.

The particle size analyser results showing that the particle size of titanium dioxide nanoparticles is present in range of 96 to 107 nm and average size of particle is 101 nm.

The zeta potential distribution results showing -9.4 mV average value for zeta potential which means negative surface charge is present and stability of nanoparticle is very low which will be enhance when these are incorporated in copolymer solution and applied on glass surface.

Particle size and zeta potential of TiO_2_ nanoparticles has been determined and results reported.

### Coating thickness

**Figure 6:**
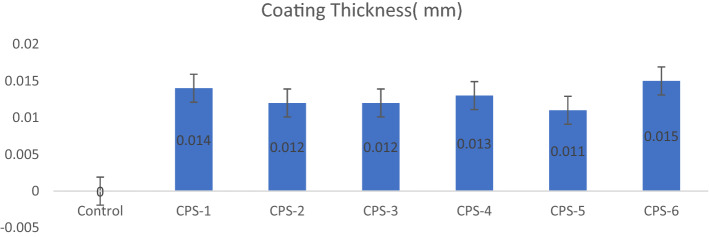
Coating thickness.

Thickness of developed coating has been measured by using coating thickness gauge and results are reported in Table 4 and Fig. 6.

**Table 4:** Thickness of Coatings.

Sr.No	Polymeric material	Coating thickness (mm)
1	Control	0.00
2	CPS.1	0.014
3	CPS.2	0.012
4	CPS.3	0.012
5	CPS.4	0.013
6	CPS.5	0.011
7	CPS.6	0.015

### Scanning electron microscopy of coating materials

**Figure 7:**
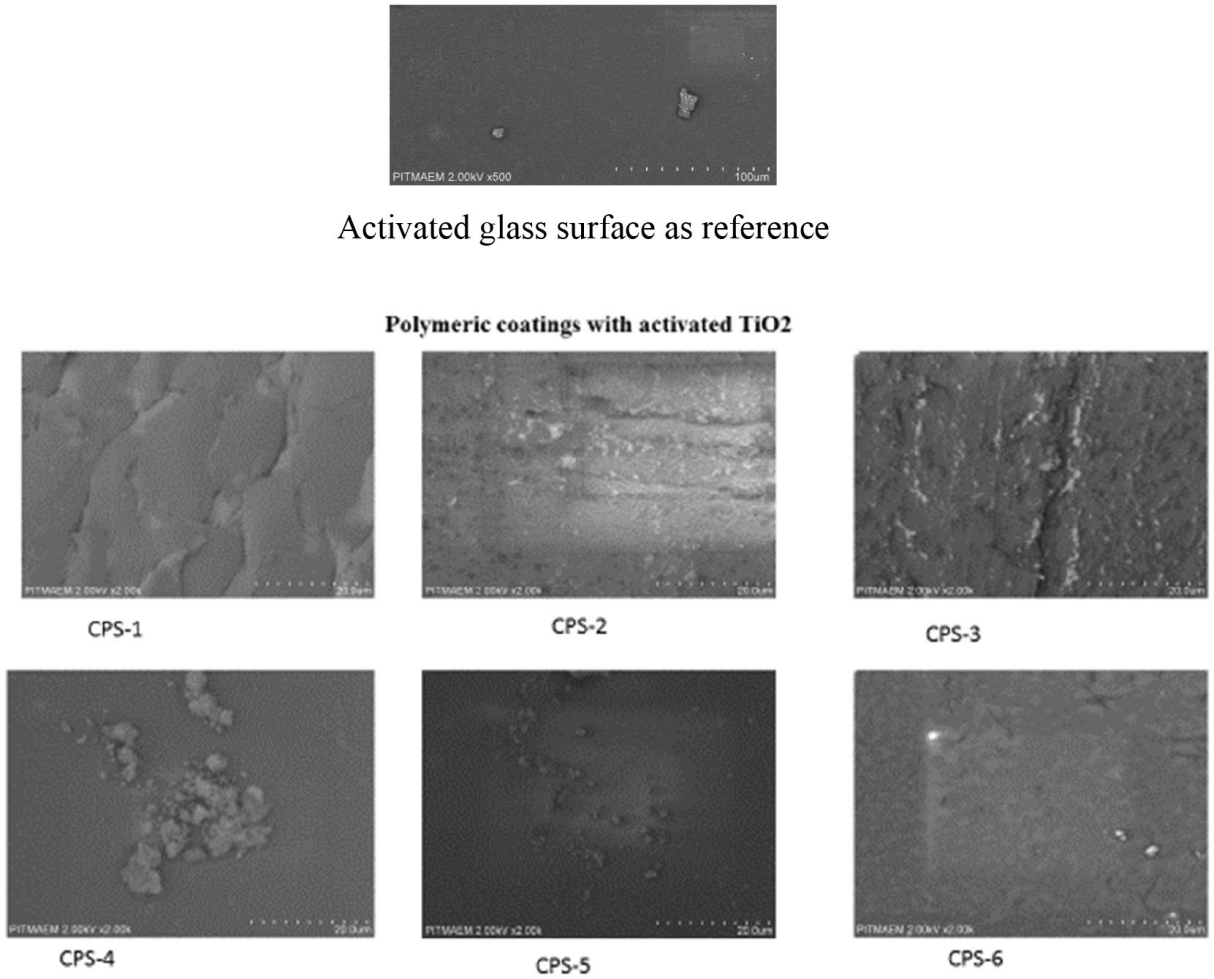
SEM analysis of coated samples.

SEM Analysis for developed acrylate-based coating composition containing MMA, 2EHA, HEMA and TiO_2_ Agglomerates etc. has been performed. As previously mentioned, TiO_2_ powder of nano sized doped with copolymer shown in the form of micron-sized agglomerates. Different compositions of acrylate coatings were examined; the images represent each of the surfaces. Surface activation can be seen through (SEM) pictures and the smooth surface of modified TiO_2_ based polymeric material as single-layered coating. On the basis of these we can conclude that titanium dioxide based coatings have been deposited on glass surface which can be further efficient for the photo-degradation/cleaning efficiency. The smooth and without cracks coating is thermoset and stable as shown in Fig. 7.

### Contact angle

**Figure 8:**
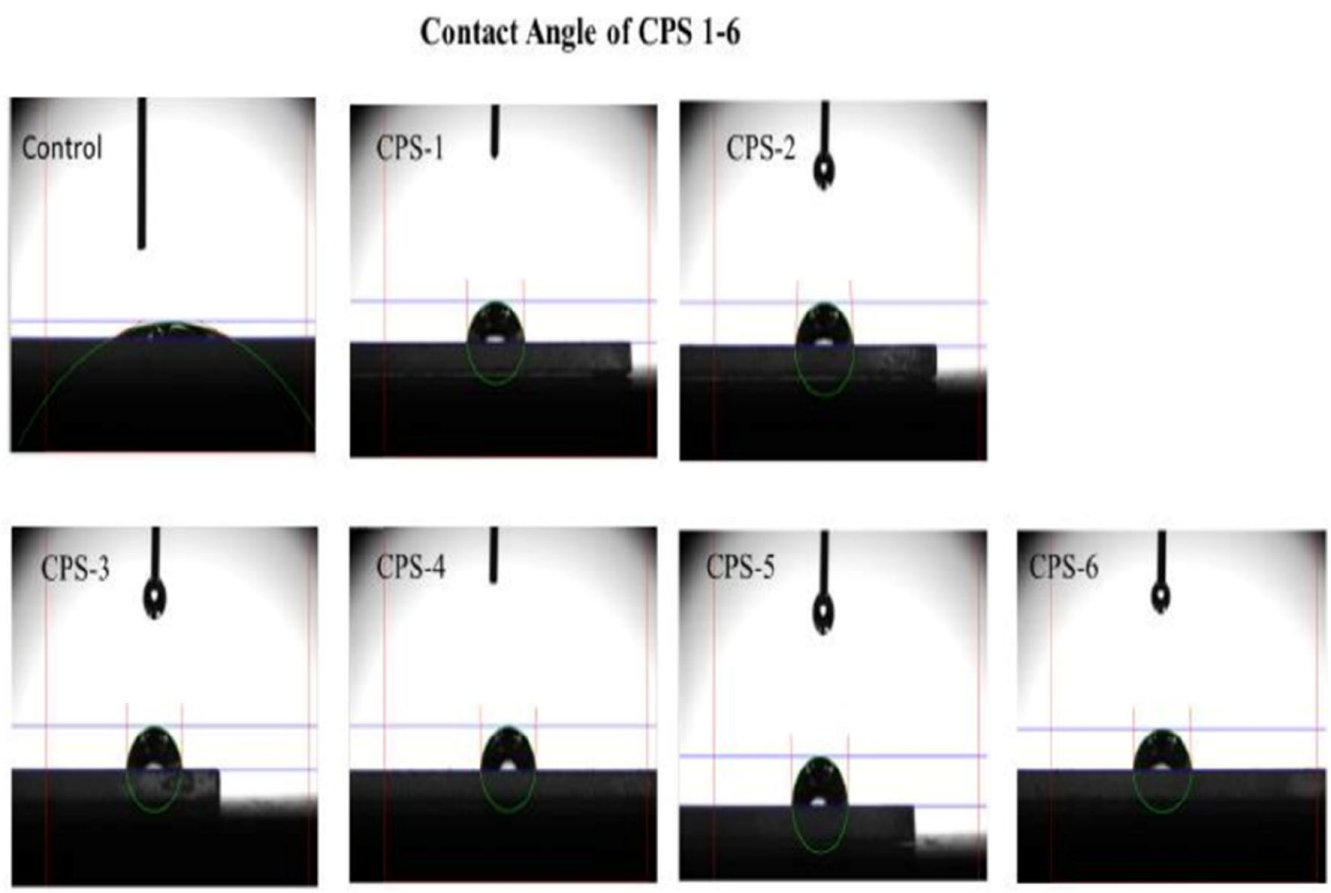
Contact angle of coated slides.

**Figure 9:**
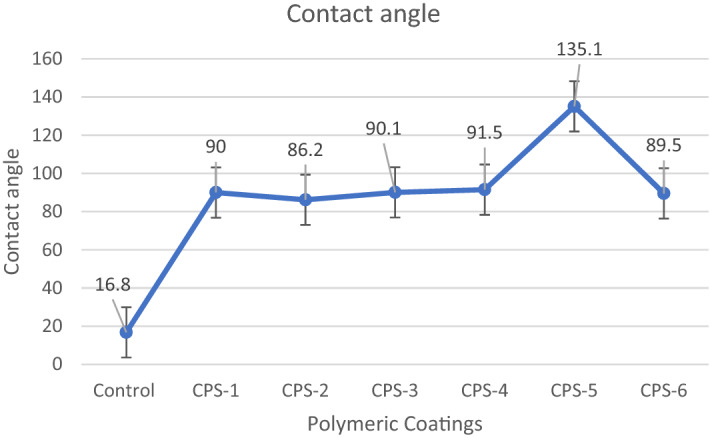
Graphical representation of contact angle.

The contact angle of water droplet on the films under ambient conditions using the DSA30 Kruss Hamburg, Germany indicates its hydrophobic nature. The coating surface was placed and levelled on the test cell between the light source and microscope. Then 10µL water droplet was deposited onto the coating surface through a syringe. After the liquid drop reaches its equilibrium state, its digital image was recorded and the contour fitted by software. The smooth surface contact angle is very low which is improved by coatings after surface modification and nanoparticles incorporation after activation with an acid and temperature role, as a result, coated glass shows no holes and cracks which means that the transparent hydrophobic coating on glass substrate show excellent surface durability. Results shown in Figs. 8, 9 and Table 5.

**Table 5:** Contact angle of water on glass slides CPS1-6.

Sr.No	Co-polymer	Contact angle
1	Control	16.8
2	CPS-1	90.0
3	CPS-2	86.2
4	CPS-3	90.1
5	CPS-4	91.5
6	CPS-5	135.1
7	CPS-6	89.5

### DSC-TGA analysis

**Figure 10:**
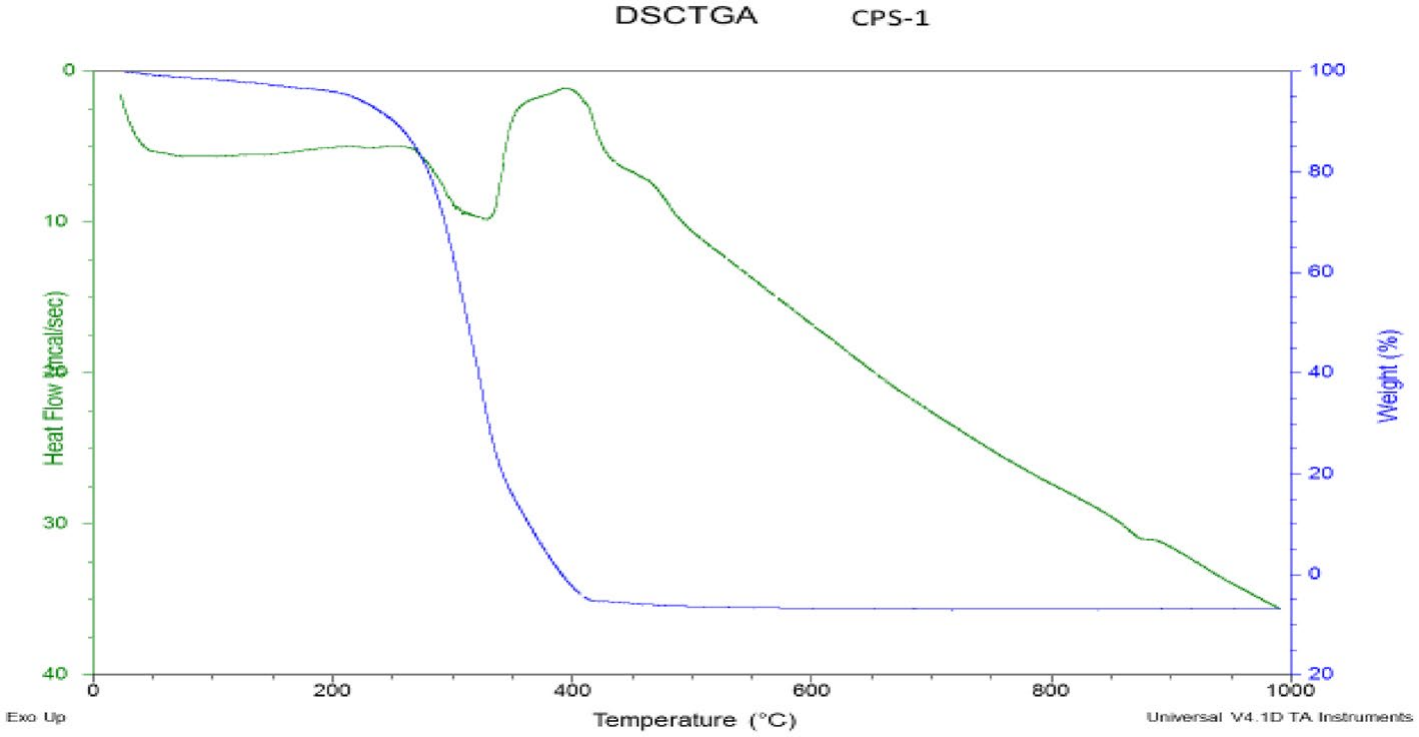
DSC/TGA of CPS-1.

**Figure 11:**
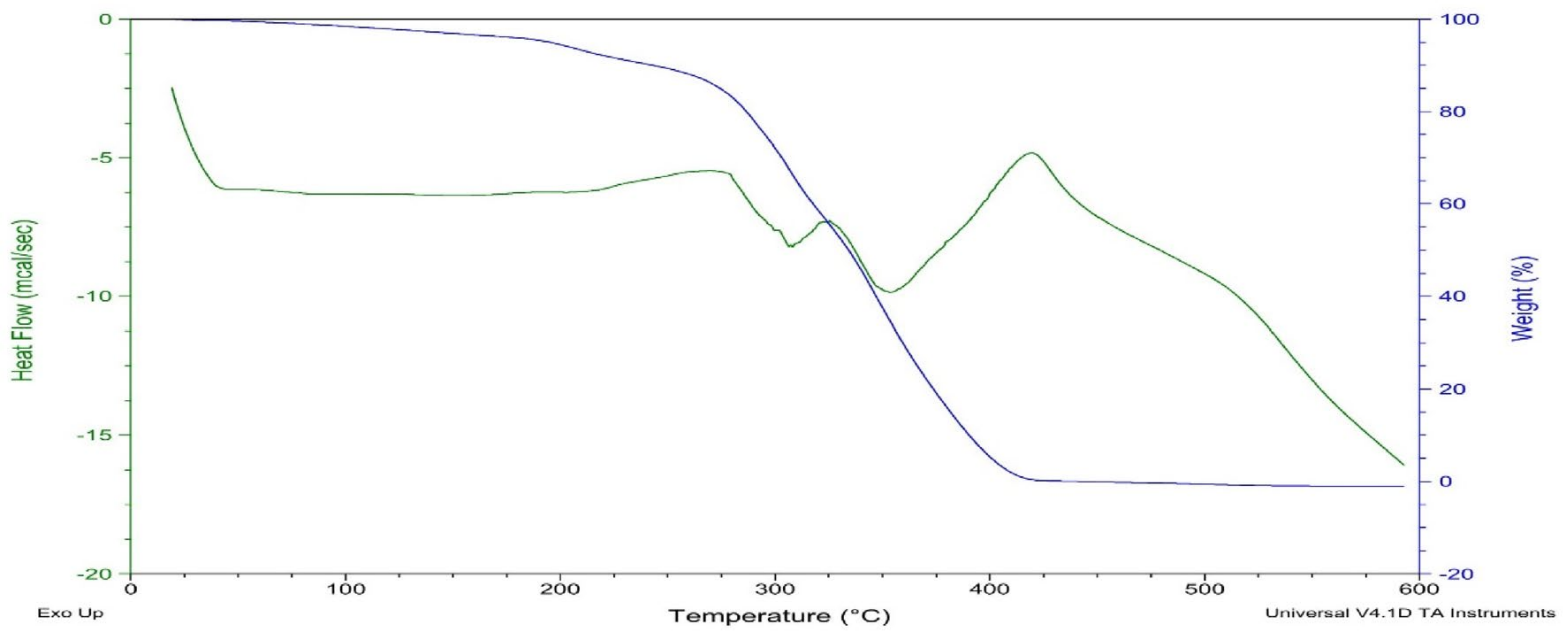
DSC/TGA of CPS-5.

TGA analysis was conducted by using SDT Q600 V8.0 Build 95on the acrylate-based coating material along with activated TiO_2_ to copolymer and to detect the thermal degradation upon addition of functionalized TiO_2_. Results revealed that the developed acrylate-based coatings are thermoset and thermal degradation occur at approximate 300 °C. The TG curves in Fig. 11 show decreasing mass % with increasing temperature and evaporation of organic solvents. By the addition of TiO_2_ in copolymer increases the thermal stability, thus increasing the thermal degradation temperature which is due to the presence of ionic bonding between TiO_2_ and the polymeric chains, and ionic cross-linking formed by functionalization. Due to the intermolecular attractions, particles are present in the form of agglomerates, (Figs. 10, 11).

### Transparency of coating

**Figure 12:**
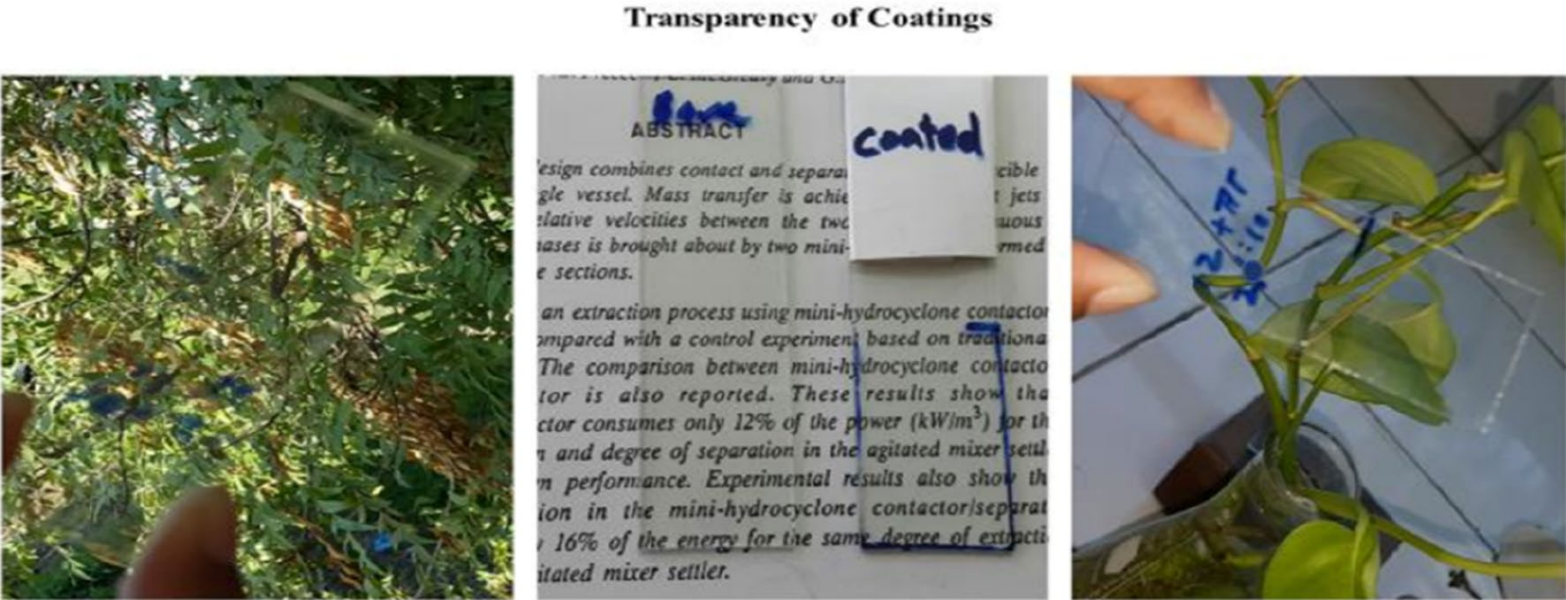
Transparency visualization of polymeric coatings.

**Figure 13:**
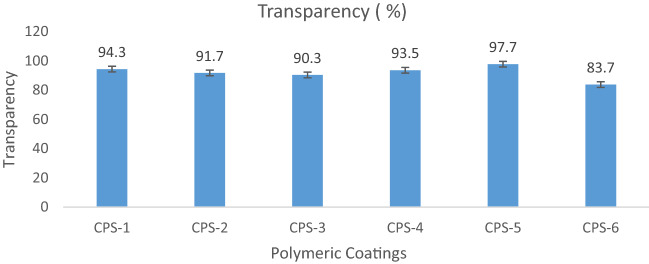
Graphical representation of coatings transparency.

In Accordance with UV transmittance data in visible range, the Coated glass slides showing transparency results of more than 97% in the wavelength range of 400–800 nm which have indicated more transparency of coating surface in visible region as compared to uncoated glass. Fog occurs in cold environment and vapours condense in the form of droplets on the glass surface which cause scattering of incident light and as a result surface becomes translucent or foggy giving poor optical performances. These polymeric Coatings are an easy approach towards anti-fogging activity. Due their high transparency these coating is best to use as protective coating on vintage things in museum. In comparison with uncoated glass, modified polymeric coating on the glass substrate recorded an average transparency of 97.5% instead of 94.3% within the wavelength range of 400–800 nm which is an indication of transparency in the visible region. From previous reports, several super-hydrophobic and hydrophobic coatings possess low transparency because of higher contact angle and roughness of surface. In general, transmittance is inversely proportional to the roughness of surface. The transparency decreases with increasing roughness. The developed polymeric coating is a single-phase coating and its transparency is not affected by the surface roughness indicated in Figs. 12, 13 and Table 6.

**Table 6:** Transparency of polymeric coatings.

S#	Polymeric coatings	Wavelength (nm)	Transparency (%)
1	Bare glass slide	400–800	93.3
2	CPS-1	400–800	94.3
3	CPS-2	400–800	91.7
4	CPS-3	400–800	90.3
5	CPS-4	400–800	93.5
6	CPS-5	400–800	97.7
7	CPS-6	400–800	83.7

### Self-cleaning analysis

Self-cleaning of coating was analysed for further specification of our hydrophobic glass.as shown in Fig. 15, the mud layer does not adhere and was expelled from modified polymeric coating. It was suggested that the coating surface has relatively low surface tension than the adhered mud; as a result, the mud is suspended above the surface. Mud is cannot be removed through simple water injection and mechanical vibration because their low force. The obtained result shows the mud adhesion left the dirt streaks on the bare glass surface and resulting to transparency degradation. Further, methyl red, methyl orange and potassium dichromate solution were dropped onto the coated glass slide surfaces. The glass samples were tilted at an angle of ~ 22° and distilled water droplet was dropped on all above slides and observed the rolling off behaviour and self-cleaning as compared to uncoated glass slide.

**Figure 14:**
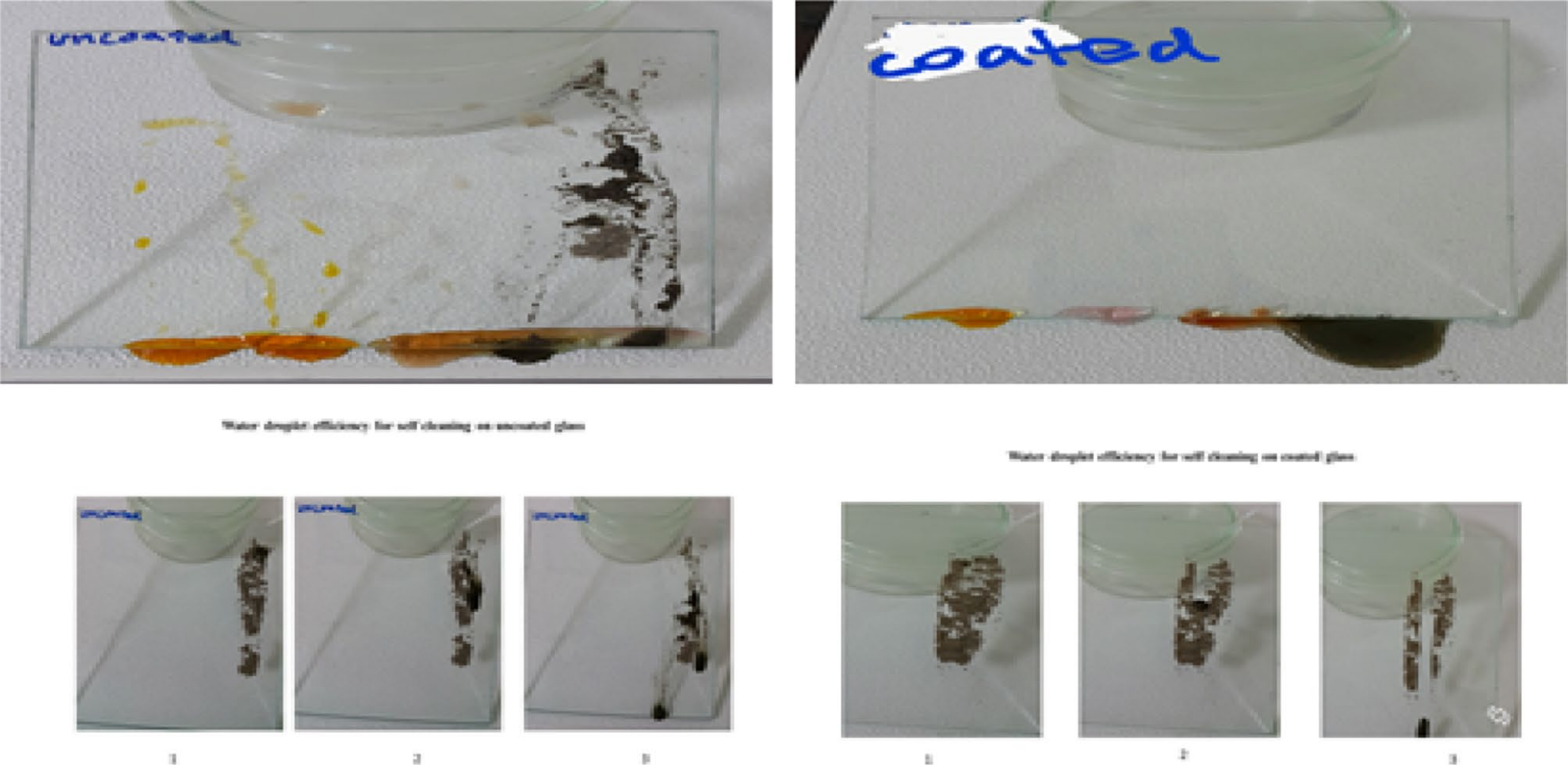
Self-cleaning efficiency of coatings.

Figure 14 above showing the self-cleaning activity of coated glass and uncoated glass against Mud, solution of methyl red, Methyl orange and K_2_Cr_2_ O_7_. All the applied liquids do not adhere to the polymeric coated surface and the liquids are removed by a sliding action. There are no dirt lines left on the coated surface showing excellent self-cleaning action. Further the lower surface tension of coating is one of the key factors for the average transmission of light before and after prolonged outdoor exposure. The suspension of applied liquids on coating surface reveals lower surface tension. In contrast to coated glass, uncoated glass failed to remove the applied liquid even at tilting angle of approximately 60°. Which cause lower transmission and thus transparency is not achieved nor self-cleaning due to spread of liquids and dirt on surface due to higher adhesion with surface of uncoated glass.

### Anti-fogging behaviour of coatings

**Figure 15:**
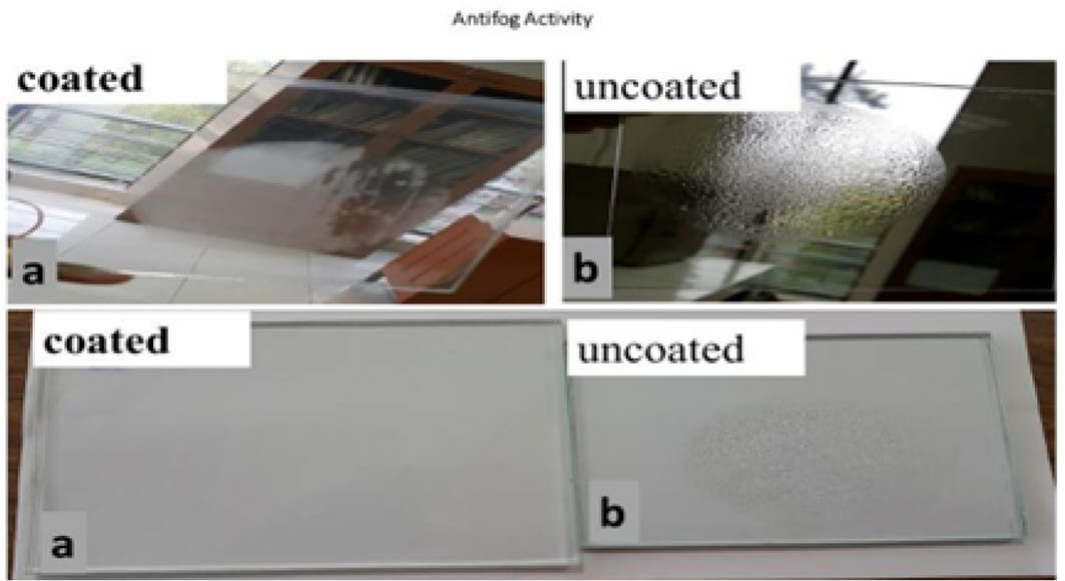
Anti-fog activity of (**a**) Coated glass (**b**) uncoated glass.

The anti-fog activity determination of bare and the coated glass was performed by placing these specimens on boiling water of 120–130 °C for 15 min. After that these were investigated by haze development and tiny water droplets. The results recorded using mobile camera which depicts that condensed droplet formed on plain and coated glass with modified polymeric coating material. The results obtained revealed that minute condensations seem on the coated glass after condensation where else plain glass slide was shielded by massive globules. The minute fog precipitations absolutely disappeared from coated glass subsequently 5 min at room temperature. The droplets persist on the uncoated glass after 20 min as shown by Fig. 15.

### Cross hatch test

**Figure 16:**
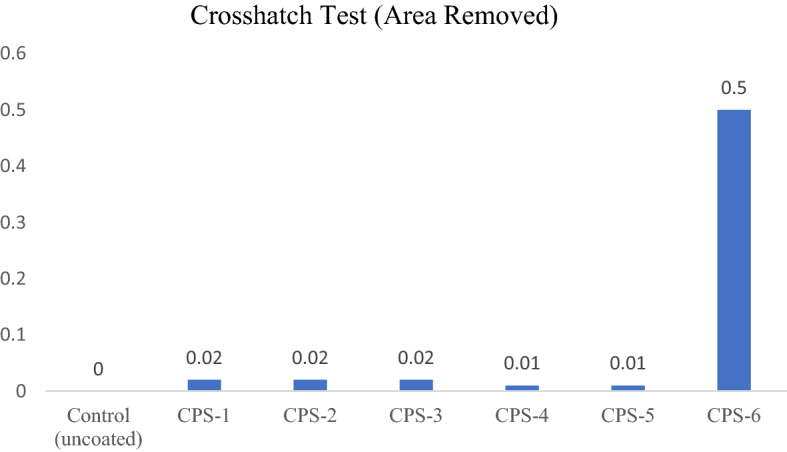
Adhesion of coating.

This method helps in determining of coating resistance from separation from its applied substrate when incisions are made as far as the substrate. This test is independently carried out on each layer with a view to determining the characteristics specific to each of them. Figure 16 show different adhesion strengths of different polymeric composition. Cross hatch tests of different polymeric composition layers. Which shows that polymeric compositions fall in range of 5B and 4B. From above fig it is obvious that addition of NPs. giving much better results than other compositions as given in Table 7 and Fig. 16.

**Table 7:** Adhesion of coatings.

Poly acrylate composition	Crosshatch test (area removed)
Control (uncoated)	0
CPS-1	0.02
CPS-2	0.02
CPS-3	0.02
CPS-4	0.01
CPS-5	0.01
CPS-6	0.5

### Shelf life of coating material

Polymeric material was applied on shield and placed in a high efficiency electric oven at 105 °C for one month no cracks appear which shows that this coating material has a shelf life more than one year.

## Conclusion

In present research a novel transparent hydrophobic surface of modified polyacrylate with TiO_2_ nanoparticles coating has been achieved by using a simple and easy fabrication process at room temperature. The coated glass surface has achieved WCA as high as 135.1° and transparency above 97% in visible region. Further theses applied coatings exhibits excellent self-cleaning properties in indoor and outdoor environment. After persistent open-air contact, coated glass slides unveils about 97% transparency, indicating its excellent self-cleaning for outdoor applications, also displays a great anti-fogging behaviour in addition, the acrylate based copolymers compositions (Cps 1–6) were successfully synthesized and TiO_2_. Water contact angle of bare glass slide is of 16° while coated surface improved to 135.1° when surface of glass is cleaned and activated with propanol. Contact angle with different acrylate copolymers coating shown in table above. These are transparent coating which can be used easily on glass substrate by dip and spray methods. Further the coatings are transparent and UV protected coatings. Results revealed that an environmentally friendly and cost-effective coating can be developed which can be used on window glass and automobile windscreen because of transparency and self-cleaning, antifogging activity.

In terms of self-cleaning property, the coating completely expels the mud, dilute solution, methyl red methyl orange and Pot. dichromate dye. Prodigious anti-foging performance of modified coated specimen has presented that tiny droplets on the coating surface are completely disappears after 7 min at ambient temperature. So such type of coatings can easily be used for outdoor and indoors in play-lands, hospitals offices etc.

## Supplementary Information

Supplementary Information.
