# Supplementary material for: Transparent, self-cleaning, scratch resistance and environment friendly coatings for glass substrate and their potential applications in outdoor and automobile industry
Source: Sci Rep. 2021 Oct 20;11:20743. doi: 10.1038/s41598-021-00230-9 (PMC8528850; doi:10.1038/s41598-021-00230-9)
Supplement: Supplementary file 1 — Supplementary Information. [file 41598_2021_230_MOESM1_ESM.docx]

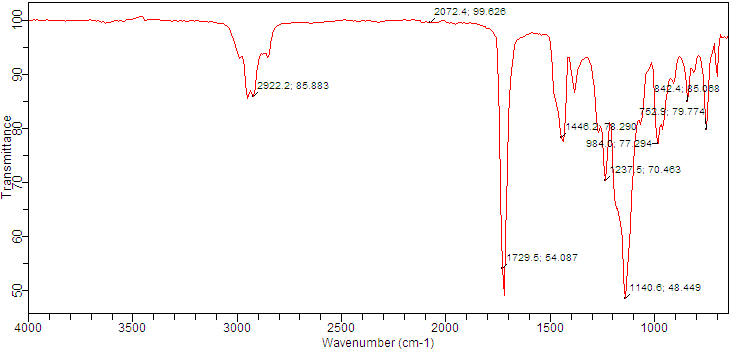


Figure-1: FTIR Spectra of CPS-1


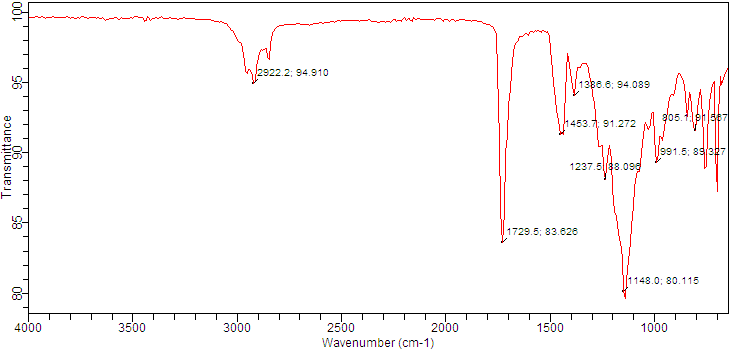


Figure-2: FTIR Spectra of CPS-2


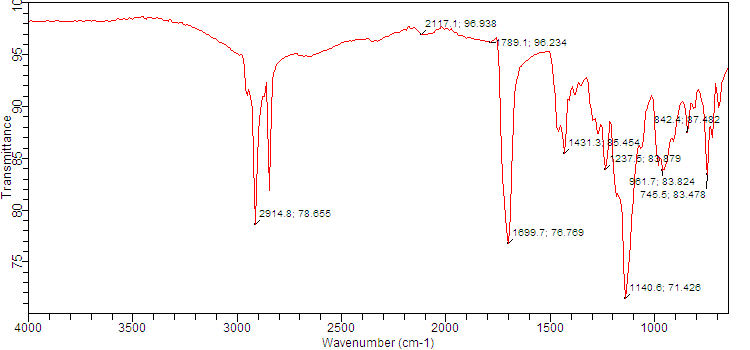


Figure-3: FTIR Spectra of CPS-3


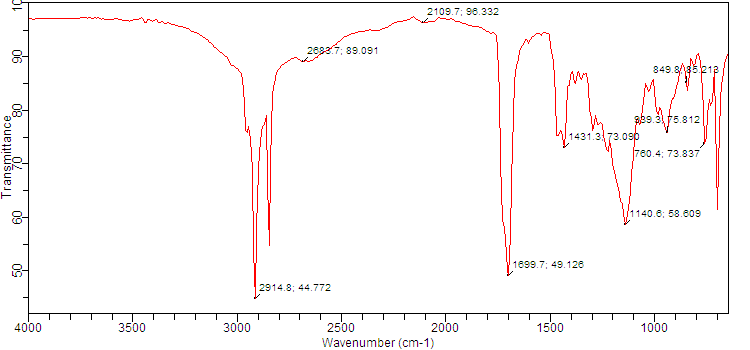


Figure-4: FTIR Spectra of CPS-4


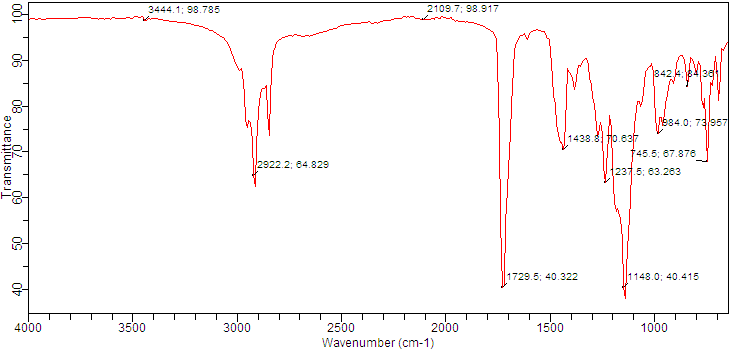


Figure-5: FTIR Spectra of CPS-5


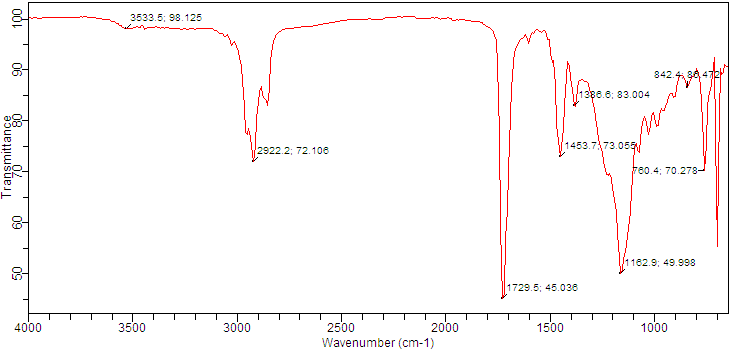


Figure-6: FTIR Spectra of CPS-6


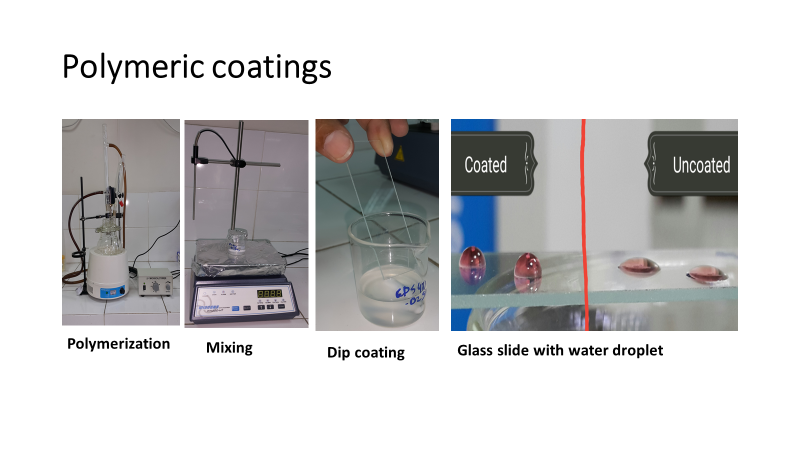


Figure -7: Coating Process
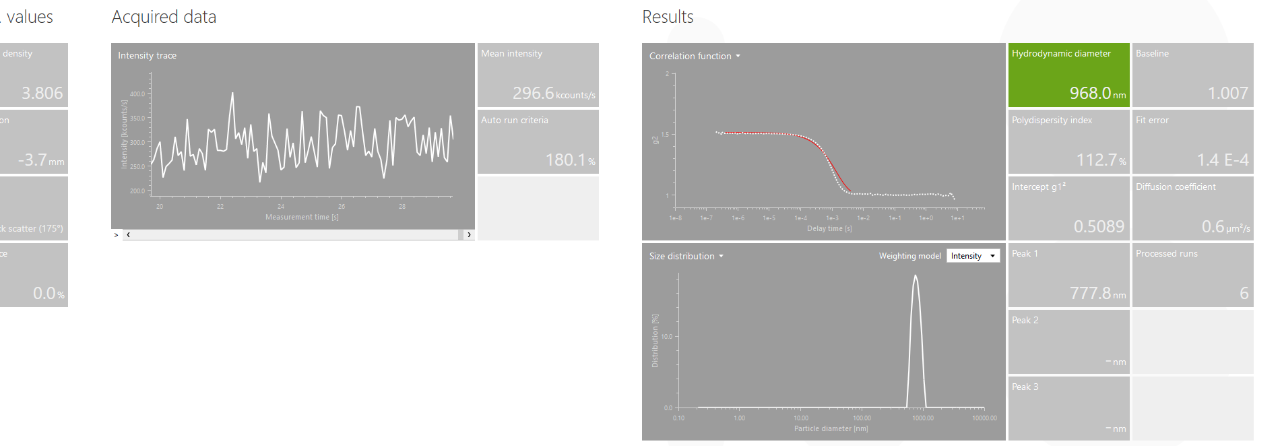


Figure-8: Particle Size of TiO2 Nps.


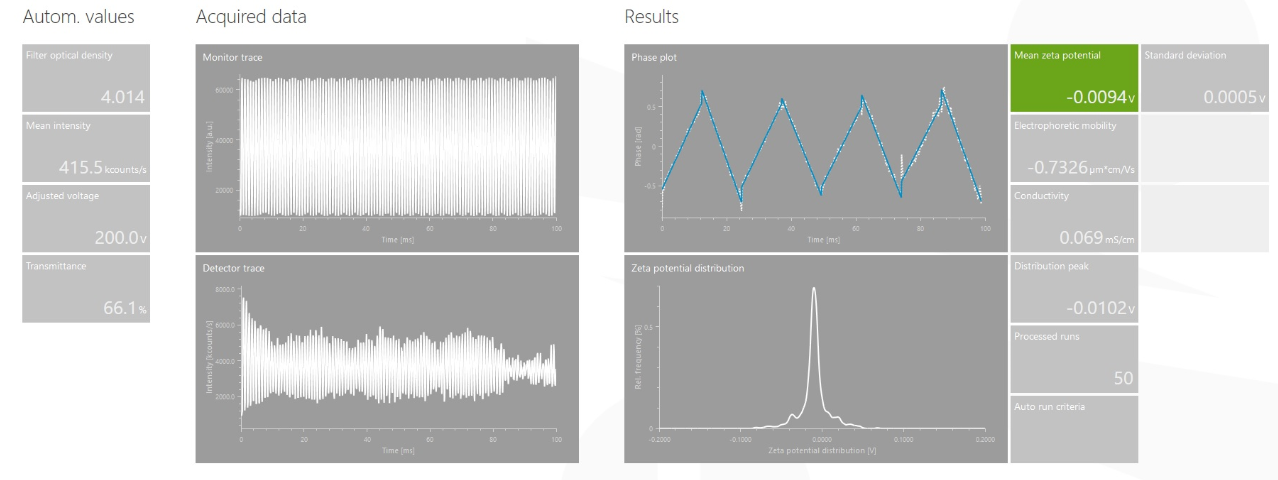


Figure-9: Zeta Potential of TiO_2_ powder.
